# Functional Diversity and Engineering of the Adenylation Domains in Nonribosomal Peptide Synthetases

**DOI:** 10.3390/md22080349

**Published:** 2024-07-29

**Authors:** Mengli Zhang, Zijing Peng, Zhenkuai Huang, Jiaqi Fang, Xinhai Li, Xiaoting Qiu

**Affiliations:** College of Food Science and Engineering, Ningbo University, Ningbo 315800, China; 18096770590@163.com (M.Z.); 216003328@nbu.edu.cn (Z.P.); hzhenkuai@163.com (Z.H.); fjq18758340317@163.com (J.F.); l15053937565@163.com (X.L.)

**Keywords:** adenylation domain in nonribosomal peptide synthetase, substrate-specificity codes of the adenylation domain, interrupted adenylation domain with methylase activity, nonribosomal peptide synthetase engineering targeting the adenylation domain

## Abstract

Nonribosomal peptides (NRPs) are biosynthesized by nonribosomal peptide synthetases (NRPSs) and are widely distributed in both terrestrial and marine organisms. Many NRPs and their analogs are biologically active and serve as therapeutic agents. The adenylation (A) domain is a key catalytic domain that primarily controls the sequence of a product during the assembling of NRPs and thus plays a predominant role in the structural diversity of NRPs. Engineering of the A domain to alter substrate specificity is a potential strategy for obtaining novel NRPs for pharmaceutical studies. On the basis of introducing the catalytic mechanism and multiple functions of the A domains, this article systematically describes several representative NRPS engineering strategies targeting the A domain, including mutagenesis of substrate-specificity codes, substitution of condensation-adenylation bidomains, the entire A domain or its subdomains, domain insertion, and whole-module rearrangements.

## 1. Introduction

In the natural environment, a peptide biosynthesis mechanism exists that does not rely on ribosomes. This process is frequently observed in bacteria and fungi and is capable of catalyzing the assembly of diverse natural peptides [1]. There are more than 500 types of monomers that have been discovered to be incorporated in such peptides [2], including amino acids (proteinogenic and nonproteinogenic amino acids), α-hydroxy acids, α-keto acids, and other types of acyl monomers [3,4,5]. This pathway is commonly referred to as the nonribosomal peptide synthetase (NRPS) pathway, which is a peptide assembly line composed of a sequence of monomer-specific mega-enzyme units, distinct from other types of ribosome-independent peptide synthesis pathways, such as those utilizing tRNA-independent acyl-AMP-ligases, ATP-grasp-ligases, tRNA-dependent cyclodipeptide synthases, and Fem-like ligases, as well as hybrid pathways for producing peptide bond containing secondary metabolites [6].

Marine microorganisms represent a significant reservoir of biological diversity on Earth, characterized by extensive genetic variability and the capacity to produce biologically active natural products. Research has demonstrated the presence of NRPS pathways in a variety of marine organisms [7,8], highlighting the potential of marine microorganisms as an important source of novel nonribosomal peptides (NRPs) metabolites [7,9].

Differences in substrates, the number of domains, and order in NRPS have led to the discovery of various peptide products synthesized through these pathways. Although certain NRPs can be toxic [10], the majority are bioactive secondary metabolites. Additionally, some NRPs offer unique advantages for human health. Many existing drugs are derived from NRPs or compounds biosynthesized by hybrid NRPS-polyketide synthase (PKS) pathways. In 2000, prepatellamide A, a cyclic peptide with cytotoxicity against P388 murine leukemia cell lines was isolated from the cytotoxic extracts of the ascidian *Lissoclinum patella* [11]. Additionally, two linear tetrapeptides, padanamides A and B, were identified in the crude extract of a *Streptomyces* species obtained from marine sediment, both exhibiting cytotoxic activity against Jurkat T lymphocyte cells (ATCC TIB-152), with padanamide B being more potent [12]. More recently, three hybrid NRPS-PKS metabolites, referred to as Flavipesides A−C, were discovered to be produced by a particular strain of filamentous fungus, *Aspergillus flavipes* 164013, which was isolated from the sponge Dysidea species found on Yongxing Island in the South China Sea. These metabolites can inhibit pancreatic lipase, potentially preventing hyperlipidemia and obesity without showing toxicity to normal cells [13]. A significant number of antiviral, antifungal, and antitumor drugs, as well as immunosuppressants, are NRPs, with 70% of these NRPs discovered in marine microorganisms [14]. 

**Figure 1:**
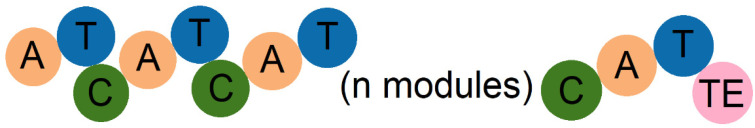
The typical domain arrangement of NRPS. Adenylation (A) domain, thiolation (T) domain, condensation (C) domain, and thioesterase (TE) domain are represented in orange, blue, green, and pink circles, respectively. Note: The module illustrated in the figure refers to a unit that comprises the three core domains C, A, and T (the initiation module, shown on the left, consists of A and T domains only, while the termination module, illustrated on the right, contains C, A, T and TE domains).

NRPS is typically composed of multiple modules, the units that conduct the assembly of building blocks for generating NRP [15] (Figure 1). The first synthesis module in the assembly line is referred to as the initiation module, usually consisting of an adenylation (A) domain and a peptidyl carrier protein (PCP) domain, also known as the thiolation (T) domain. A domain specifically activates the acyl monomer substrate and subsequently loads the activated substrate to the 4′-phosphopantetheine (Ppant) arm in the T domain, which enables the monomer substrate to be loaded for connection with another monomer bound to the T domain in the downstream module, which is referred to as the elongation module. The elongation modules, located downstream of the initiation module, sequentially add monomers to the growing peptide chain and typically contain three core domains: A domain, T domain, and condensation (C) domain that catalyzes the formation of peptide bonds between T domain-bound monomers and peptides. In elongation modules, the core domains are typically arranged in a C-A-T order for the sequential incorporation of amino acids or other acyl monomers in order (Figure 1). As the peptide chain elongates, substrates move from the initial module to the termination module as the peptide chain grows. At the end of NRP synthesis, the release of the final product is typically catalyzed by a thioesterase (TE) domain located at the C-terminus of the termination module through hydrolysis or cyclization [16,17,18,19,20,21,22]. In addition to the core domains mentioned above, some NRPSs also possess additional optional domains such as the epimerization (E) domains in tyrocidine synthetases TycA and TycB from *Bacillus brevi* [23], the reduction (R) domain in NRPSs involved in soramycin synthesis, and the oxidation (Ox) domain involved in bleomycin synthesis [24]. These domains also play essential roles in NRP synthesis and release, contributing to the structural diversity observed in NRPs.

The organization of NRPS into multiple modules typically follows the principle of colinearity, where the specificity of substrates for a series of modules corresponds to the sequence of the final product. Within the NRPS assembly line, the A domain serves as the first domain required to load substrates for generating peptide chains, acting as the primary gatekeeper of substrate specificity in NRPS. Each A domain generally exhibits specificity towards a particular substrate [25], although some studies have indicated that certain types of A domains can specifically recognize multiple substrates [26]. The substrate specificity of various A domains has been biochemically characterized, demonstrating the dominant influence of the sequence of the A domain on the final peptide product sequence [27,28]. Advances in genomic sequencing and functional analysis of adenosylase have gradually improved the accuracy of bioinformatics tools for predicting substrate specificity of the A domain [29]. Various prediction methods, such as the AdenPredictor extra tree machine learning model, have been developed to improve the accuracy of substrate specificity prediction for A domains, facilitating research in this area. [30,31,32]. Although bioinformatics prediction tools are not always accurate, especially for rare substrates such as nonproteinogenic amino acids and several unique monomers derived from marine organisms [33], they can serve as auxiliary tools in guiding the discovery of novel NRPs [34].

The NRPS A domains have been identified in more than 90,000 protein sequences, predominantly found in actinobacteria, proteobacteria, bacilli, fungi, and cyanobacteria, as reported by the InterPro database [34]. This database serves as a valuable resource for researchers in screening target strains and expediting NRPS engineering studies to generate a wider array of novel NRPs with varied functionalities. Historically, the research on NRP engineering efforts has concentrated on the A domain, offering numerous avenues for NRP customization. In this article, based on a brief overview of the functions of A domains in terms of the mechanism of substrate adenylation and other auxiliary functions, several representative types of engineering strategies of A domains, including mutagenesis of substrate-specificity codes, the substitution of the C-A bidomain, the entire A domain or its subdomains, domain insertion, and rearrangements at the level of the whole module, are discussed in detail, providing insights for further comprehensive research in this field. 

## 2. Major Function and Functional Diversity of A Domains

### 2.1. Substrate-Activation Function of A Domain

Studies on the A domain-catalyzed incorporation of monomer substrates into peptide chains can be categorized into two primary areas: one is the substrate recognition mechanism of the A domain, while the other is the mechanism of substrate adenylation catalyzed by the A domain.

#### 2.1.1. Substrate Recognition Mechanism of A Domain

The characteristics of specific recognition of substrate in the A domain can be identified by the substrate-specificity code consisting of a series of key residues. Two decades ago, the relationship between the active site of the A domain and substrate specificity was revealed based on the crystal structure of the first A domain of Gramicidin S synthetase 1 (GrsA-A) and its complex with a substrate, leading to the introduction of the concept of substrate-specificity codes, also known as the substrate binding pocket [27,35]. Table 1 lists the 10 substrate-specificity codes of several A domains that correspond to L-α-amino acids. It demonstrates that A domains recognizing the same substrate exhibit highly similar substrate-specificity codes [35], which can exhibit variability within a certain range, indicating the diverse recognition modes of L-α-amino acids.

##### Amino Acid Recognition Mechanism of A Domain

Considerable research efforts have been dedicated to the production of non-natural NRPs via biosynthesis, involving the incorporation of substrates tailored to meet the requirements for the synthesis of final products. Noteworthy observations have emerged from these investigations. For example, within the α-amino acid substrate-selective A domain, the Asp235, and C-terminal Lys residue are highly conserved, as they interact specifically with the α-amino and α-carboxylate groups of the α-amino acid, respectively, to define its positioning and orientation during catalysis. The remaining eight specificity-coding residues are involved in the recognition of side chains of α-amino acid substrate. A comprehensive database has been established by gradually accumulating data on the relationship between enzyme sequences and substrate specificity, which aids in enhancing the accuracy of predicting substrate specificity for additional A domains [36,37,38].

**Table 1:** Several typical substrate-specificity codes of A domains correspond to L-α-amino acids.

	Substrate	235	236	239	278	299	301	322	330	331	517	Reference
GrsA	Phe	D	A	W	T	I	A	A	I	C	K	[39]
TycA	Phe	D	A	W	T	I	A	A	I	C	K	[39]
SrfA-B	Val	D	A	F	W	I	G	G	T	F	K	[35]
CcsA-M9	Val	D	A	W	M	F	A	A	V	L	K	[39]
GrsB	Val	D	A	F	W	I	G	G	T	F	K	[39]
CepA	Tyr	D	A	S	T	V	A	A	V	C	K	[39]
TycC	Tyr	D	A	L	T	T	G	E	V	V	K	[39]

##### Keto Acid Recognition Mechanism of A Domain

Previous studies have predominantly focused on amino acid-selective A domains, but various types of A domains have been identified to recognize other classes of acyl substrates. For instance, the A domain of StsA (StsA-A) specifically recognizes α-keto acid. StsA-A employs a unique mechanism to differentiate between α-keto acids, α-amino acids, and α-hydroxy acids, favoring the adenylation of α-keto acid and transferring the adenylated intermediate to T domain. The T domain then transports the α-keto acid to the ketoreduction (KR) domain to generate an α-hydroxy acid monomer through stereoselective reduction of the α-keto group. Subsequently, the α-hydroxy acid monomer-loaded T domain moves to the downstream C domain within the module for condensation, leading to the formation of ester bonds (or directly enters the C domain of the downstream module in the case of the initial module A-KR-T) [40,41]. 

#### 2.1.2. Catalytic Mechanism of the A Domain

**Figure 2:**
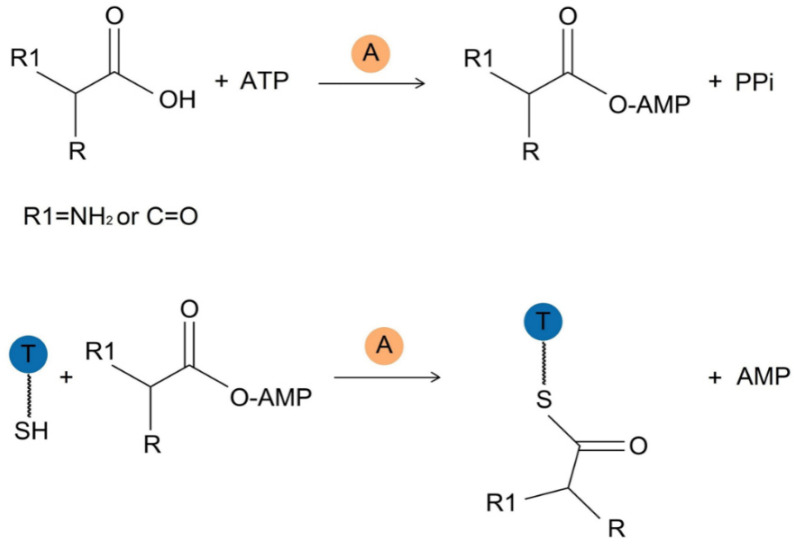
Activation and loading of substrate catalyzed by the A domain. Initially, the A domain activates the acyl monomer: substrate reacts with adenosine 5′-triphosphate (ATP) to generate the acyl-adenosine monophosphate (AMP) intermediate and inorganic pyrophosphate (PPi). Subsequently, the aminoacyl-AMP undergoes nucleophilic attack by the thiol group located at the terminus of the 4′-phosphopantetheine arm of the downstream thiolation (T) domain, leading to the formation of a thioester-bound aminoacyl-S-T domain by linking to the thiol of the phosphopantetheine arm of T domain, followed by the release of AMP.

The A domain catalyzes the activation and loading of the acyl monomer in an ATP-dependent manner. Taking amino acid substrate as an example, A domain catalyzes two sequential reactions involving the activation of amino acid to generate an aminoacyl-adenosine monophosphate (AMP) intermediate, followed by the transfer of this intermediate to the 4’phosphopantetheine (Ppant) group in the downstream T domain [42,43] (Figure 2). 

The structure of the A domain can be divided into two parts: the larger N-terminal core domain A_core_ (with a size of approximately 50 kDa) and the smaller C-terminal domain A_sub_ (with a size of approximately 10 kDa). During the catalytic reaction process, the A domain undergoes a conformational change. Initially, the A domain adopts an open conformation, where both the A_core_ domain and A_sub_ domain are in an open state to accommodate substrate and ATP. Subsequently, the A domain transitions to the catalytic conformation, leading to a shrinkage of the cavity between the A_core_ and A_sub_ domains to generate aminoacyl-AMP. Finally, the A_sub_ domain is displaced, resulting in the binding of aminoacyl-AMP to the T domain, and the A domain reverts to the open conformation [44].

Certain A domains require interaction with small-sized protein activators, such as MbtH-like proteins (MLPs), to fulfill their catalytic function effectively. MLPs, named after the MbtH protein in the mycobactin operon from *Mycobacterium tuberculosis* [45], are a class of proteins consisting of 70 amino acids. Previous research has investigated the substrate preference of the A domain of Hrm, an NRPS module of hormaomycin. When attempts to express heterologous proteins failed, it was discovered that some A domains exhibited activity only in the presence of co-expressed MLPs, highlighting the A domain’s reliance on MLPs [46,47]. Subsequently, Herbst et al. expressed and purified SlgN1, a 3-methylaspartate-adenylating enzyme with an N-terminal MLP. They designed an expression construct for SlgN1-ΔA_sub_, a fusion protein containing an MLPs and an A_core_ domain, by removing the C-terminal A_sub_ domain to obtain the crystal structure of this adenylation enzyme. Crystal structure analysis demonstrates that MLP is far from the active center and does not directly interact with the substrate. Mutation of Ala433 to Glu in MLP abolished the activity, and mutation of Ser23 located on the MLP interaction surface to Tyr decreased the activity. However, the activity was restored upon the addition of a complete MLP, providing the first direct evidence and functional characterization for this binding mode and supporting the significant impact of MLP on A domain activity [48]. Additionally, other studies have also shown that MLPs can enhance the activity of the A domain [45], so MLPs are necessary for the activity of some particular NRPS. This makes them promising drug targets for developing antibacterial compounds that disrupt the iron carrier of pathogens [49]. Acyl CoA synthetase and luciferase, members of the ANL adenylase superfamily like A domain, exhibit similar catalytic functions. Despite differences in the overall catalysis process of these two types of reactions, they generally follow a two-step mechanism involving ATP to form an adenylate intermediate and release pyrophosphate (PPi) to activate the substrate in the first step, followed by the release of AMP in the second step [42].

### 2.2. Auxiliary Functions of A Domains

The substrate-specificity codes of the A domain are composed of 10 elements (a1–a10), which are split into N-terminal (a1–a7) and C-terminal (a8–a10) codes. Some A domains exhibit special structures, with additional catalytic regions such as the methylation (M) domain, Ox domain, and KR domain inserted into the domain. Typically, these insertions occur between codes a8 and a9 and sometimes between a2 and a3. A domain with such interruptions is referred to as an interrupted A domain [50,51,52]. The interrupted A domain exhibits two types of functions: its intrinsic adenylation function and the function corresponding to the insertion domain. In the case of an interrupted A domain with M domain insertion, methylation modification is performed using *S*-adenosyl-L-methionine (SAM) as the methyl donor, and the inserted M domains do not accept free substrate. Adenylation occurs first to activate the substrate, and methylation takes place only after the linkage of the activated substrate to the T domain [53]. M domains in the interrupted A domains are generally divided into two main categories: those responsible for main-chain N-methylation and the other corresponding to side-chain O- or S-methylation. The type of methylation is generally denoted by subscript notation, with “b” in M_b_ referring to main-chain methylation and “s” in M_s_ referring to side-chain methylation [54]. 

**Figure 3:**
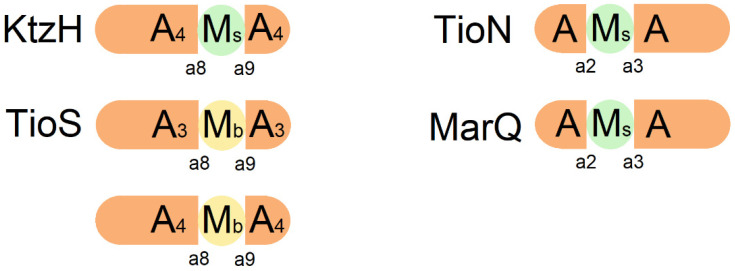
Composition of some interrupted A domains: In KtzH, an interruption occurs between a8 and a9 codes of the A domain in module 4, where an O-methylase domain is inserted [55]. In TioS, interruptions occur between a8 and a9 codes of the A domain in both module 3 and module 4, with the insertion of an N-methylase domain [53]. In TioN, an interruption occurs between a2 and a3 codes of the A domain, with the insertion of an S-methylase domain [50]. In MarQ, an interruption occurs between a2 and a3 codes of the A domain, with the insertion of an S-methylase domain [57]. M_b_: main-chain methylase domain (yellow); M_s_: side-chain methylase domain (light green).

Several studies demonstrate that the most common form of interrupted domains is the insertion between a8 and a9 codes, as the case in the NRPS module of Kutznerides. Kutznerides are cyclic hexadepsipeptides produced by the soil actinomycete *Kutzneria* sp. 744 through the action of three NRPS modules, namely KtzE, KtzG, and KtzH. The A domain of KtzH contains an interruption between a8 and a9 codes by an M domain, which is responsible for catalyzing the activation and O-methylation of L-Ser [55] (Figure 3). Studies of TioS, synthetase of the anticancer peptide thiocoraline derived from marine actinomycetes, have shown that interruptions occur in the A domains in both the third and fourth modules of TioS. The N-methylation domains are also generally inserted between a8 and a9 codes. Unlike the typical fusion of engineered proteins through flexible connecting regions, the connection region between the M and A domains is structured, thereby maintaining the normal folding of the A domain [53,56]. An example of this interruption is seen in TioN, a component of the thiocoraline biosynthetase, where the A domain is interrupted between a2 and a3 codes by an M domain, specifically denoted as TioN (A-M-A) [50]. This interrupted A domain exhibits both adenylation and methylation activities for L-Cys (Figure 3). A similar pattern of domain insertion is observed in MarQ, the maremycin biosynthetic module from a marine bacterium *Streptomyces* sp. B9173, where the M domain is also inserted between a2 and a3 codes of the A domain [57] (Figure 3). Studies have revealed that some interrupted A domains play an active role in biosynthesis, leading to the production of methylated analogs with enhanced oral bioavailability compared to the original compounds [58]. To provide a comprehensive overview of interrupted domains, a partial list of known interrupted domains has been compiled in Table 2, providing details on methylation products, interruption sites as well as methylation types. This information can serve as a reference for future research on the modification of NRP-derived drugs generated by interrupted A domains.

## 3. Engineering of A Domains

Investigating the substrate specificity of the A domain is particularly crucial because of its primary role in controlling substrate specificity during the process of NRP biosynthesis. In addition to altering substrate-specificity codes, methods such as splicing or domain substituting of NRPS modules are utilized to change the diversity of NRPS product peptides [63]. Not only individual domains but recognition subdomains and even entire modules can also be the target for substitution. These methods are demonstrated below and summarized in Table 3. Engineering modifications are mostly performed using *Escherichia coli* as the expression host, which has proved to be efficient for genetic engineering manipulations of biosynthetic gene clusters and the recombinant expression of their encoded proteins.

### 3.1. Site-Specific Mutation of Substrate Specificity Codes

Studies on site-directed mutagenesis of residues at substrate recognition sites and insertion of partial peptide segments have been carried out for a long time, leading to significant advancements in the field. For example, researchers performed mutations in the substrate-specificity codes that recognize Glu in the A domain of the initiation module responsible for lipopeptide surfactin biosynthesis from *Bacillus subtilis*. The introduction of the K239E mutation resulted in enhanced selectivity towards L-Gln, resulting in the generation of surfactin variants [64]. In another study, researchers selected eight key residues associated with substrate specificity in the A domain that specifically recognizes phenylalanine (PheA) in gramicidin S synthetase as mutation targets. Among these mutants, the mutation of W239S resulted in approximately a threefold higher preference for L-Tyr over L-Phe [65]. These achievements have laid the foundation for further studies on the substrate-specificity code mutations within the A domain. Similarly, during the study on how to integrate synthetically produced non-natural amino acids into calcium-dependent antibiotics (CDA), Thirlway et al. employed site-directed mutations to alter the substrate specificity of the A domain in the first module of CDA PS3. The substitution of Lys at position 278 with Gln (K278Q) successfully yielded a CDA variant containing glutamine. This study serves as a foundational example for future research endeavors aiming to introduce non-natural amino acids by manipulating the substrate specificity of the A domain in NRPS [66]. 

For A domains with dual or multiple substrate specificities, site-directed mutagenesis of substrate recognition residues can transform them into single-specificity A domains to generate the desired products [26]. An illustration of this process is demonstrated in the production of ohmyungsamycins (OMSs), a group of macrocyclic peptides with anticancer activity produced by a marine bacterial strain belonging to the *Streptomyces* genus. Within the biosynthesis pathway of OMS, the A domain in the second module can specifically recognize two amino acids, L-Val and L-Ile, respectively, corresponding to the formation of OMS-A and OMS-B, with OMS-A exhibiting stronger activity against cancer cells. By introducing double mutations (G299W and A322G), the yield of OMS-A was effectively increased [67]. Similarly, lyngbyatoxin (LTX) produced by marine cyanobacteria is known for its capacity to activate protein kinase C (PKC), a trait with therapeutic implications for PKC-related pathological conditions. Studies using the heterologously expressed protein of the double module NRPS LtxA in *E. coli* illustrated that the first A domain in the binary module can activate a variety of substrates, such as Val, Leu, and Ile. After site-specific mutations of Y239 and W299, substrate-specificity changes in this A domain were observed: the W299L mutation notably increased activity towards Val and Leu, while the Y239M mutation resulted in a higher specific preference for Leu [68].

### 3.2. Substitution of the A Domain

**Figure 4:**
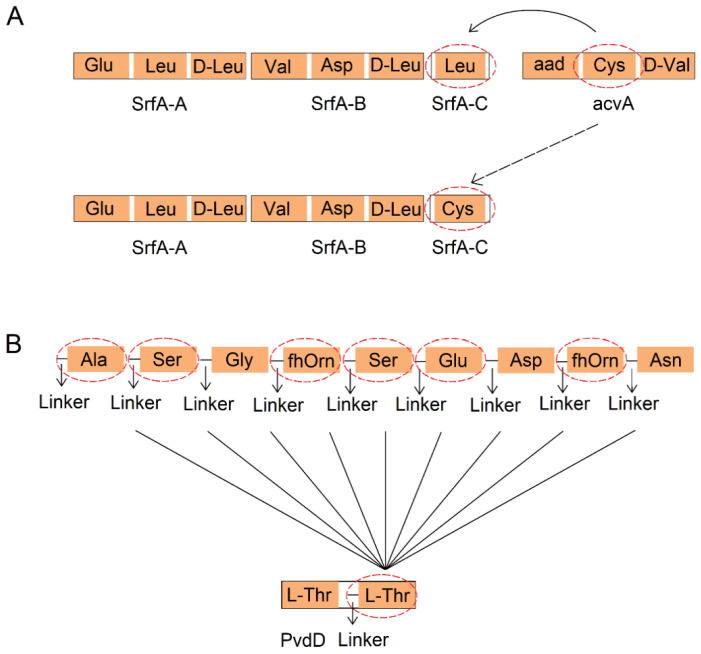
Two typical cases of A domain substitution. (**A**) The A domain in *Bacillus subtilis* SrfA-C, which recognizes Leu, is replaced with an A domain that recognizes Cys. (**B**) The A domain of the second module of PvdD that recognizes L-Thr along with the linker region is replaced with a randomly selected one from nine different types of A domains that recognize other substrates and their corresponding linker regions. Among these, substitutions of six exchanged A domains labeled with dashed circles achieved higher yields. Abbreviations: aad: δ-L-α-aminoadipyl residue, fhOrn: N5-formyl-N5-hydroxyornithine residue.

Because the A domain plays a primary role in the substrate recognition process within NRPS, this domain is usually a priority target for NRPS domain substitution. This concept was proposed and experimentally studied in the 1990s, with subsequent experiments gradually confirming the significant role of substituting either the entire A domain or a portion of it in the diversification of NRPSs [69]. Marahiel et al. substituted the A domain that recognizes Leu in SrfA-C from *Bacillus subtilis* with an A domain recognizing Cys (Figure 4A). Through this substitution, they successfully constructed a hybrid peptide synthetase with altered amino acid substrate specificity. This innovative approach led to the recombination of SrfA, enabling the synthesis of surfactin analogs with hemolytic activity in *Bacillus subtilis* for the first time [70].

Substitution of the A domain may not always yield the desired outcomes, as it can result in compromised activity, reduced peptide product yield, and abnormal expression of recombinant proteins. Over the years, researchers have been actively exploring effective strategies for the substitution of A domains. PvdD, a dual-module NRPS in *Pseudomonas aeruginosa* PAO1 responsible for incorporating the last two Thr residues into pyoverdine, has been a subject of study in this regard. By substituting the A domain in the second module of PvdD with Thr-selective A domains from different bacterial strains, researchers were able to synthesize pyoverdine successfully. Conversely, A domains that were not selective for Thr only produced minimal amounts of pyoverdine compounds, indicating a lack of expected functionality under the new environment [71,72]. Subsequent experiments involved substituting the A domain in the second module of the PvdD module, along with the linker region (an approximately 36-residue sequence extending from the C-terminus of the last helix in the C domain to the first helix in the A domain), resulting in increased yield of pyoverdine products. Furthermore, nine A domains from *Pseudomonas* species that activate other substrates were randomly selected and replaced into the second module of PvdD (Figure 4B), which led to a significant enhancement in the yields of six of these A domain-exchanged hybrids [69]. More studies are continuously attempting to optimize domain exchange methods, with progress being made in addressing some of the associated challenges.

### 3.3. Substitution of the Recognition Subdomain of the A Domain

**Figure 5:**
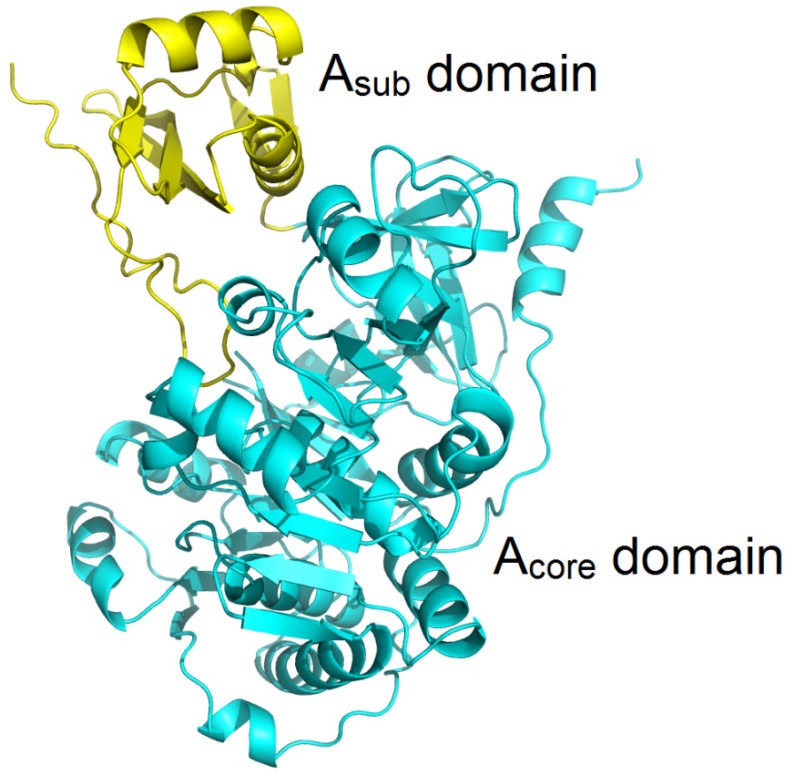
The cartoon representation of the overall structure of GrsA (PDB ID: 1AMU), phenylalanine-selective A domain of gramicidin synthetase 1, showing the subdomain and core domain of the A domain, which are represented in yellow and cyan, respectively.

Previous studies suggest that substrate-specificity codes are located within a subdomain of the A domain, referred to as the recognition subdomain (RS) (Figure 5) [73]. To modify the substrate specificity of the A domain, one can employ a technique involving the substitution of the substrate-specificity code by replacing the RS segment of the A domain. Kries et al. pinpointed the RS within the A domains of gramicidin S synthetases, GrsA and GrsB, and exchanged these subdomains into GrsA to investigate their amino acid substrate recognition patterns. Among the nine different RS substitutions, the subdomain from GrsB that recognizes Val exhibited the same amino acid-specific recognition mode in GrsA. Furthermore, through the construction of a module containing RS-substituted GrsA and GrsB1, they successfully obtained the desired product, demonstrating the feasibility of altering the substrate specificity of the A domain through subdomain substitution [74].

**Figure 6:**
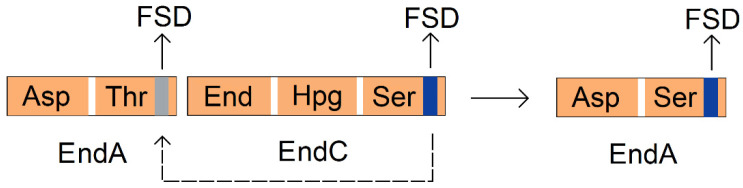
The second A domain of EndA that recognizes L-Thr. The FSD responsible for substrate recognition in this domain is replaced with the FSD from EndC that recognizes Ser, enabling the second A domain of EndA to recognize Ser. Abbreviation: Hpg: hydroxyphenylglycine.

The exchange of subdomains has a negligible impact on the overall structure of the A domain, allowing for the preservation of crucial interactions with other domains within the assembly line. Notably, the effects of subdomain exchange are more pronounced when dealing with homologous A domains that share high sequence similarities. For instance, by substituting the flavodoxin-like subdomain (FSD, containing key active site residues within the A domain) from the second A domain of EndA, which recognizes L-Thr in endracidin biosynthesis, with the FSD from EndC that recognizes L-Ser (with 88% identity and 89% similarity) (Figure 6), an endracidin variant with L-Ser replacing L-Thr was successfully obtained [75].

### 3.4. Domain Insertion 

**Figure 7:**
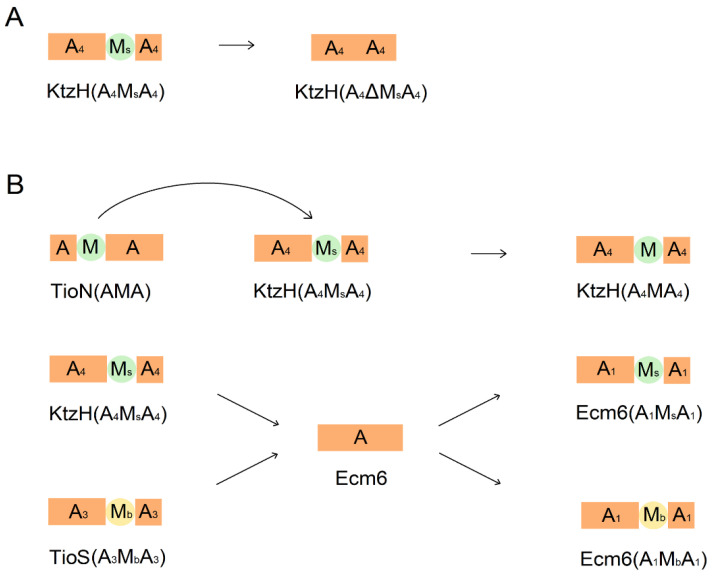
Several typical cases of sequence removal and insertion in A domains. (**A**) The wild-type A domain of KtzH is naturally interrupted. The methylase activity of this domain is abolished after its interrupted Ms domain is removed. (**B**) Replacing the interrupted M_s_ domain in KtzH with the interrupted M domain from TioN yields an A domain exhibiting both methylation and adenylation functions; inserting the interrupted M domains from KtzH and TioS into the uninterrupted A domain of Ecm6 results in the generation of corresponding A domains with dual functions of methylation and adenylation.

Interrupted A domains, as described in 2.2, are a distinct form of A domains that can be generated by removing the insertion domains, exchanging insertion domains, or artificially disrupting uninterrupted A domains to achieve diversity in the resultant peptide products. Shrestha et al. removed a portion of the M_S_ domain (The domain previously referred to as the M_H_ domain) between a8 and a9 codes of the interrupted A domain in KtzH, thereby designing a new uninterrupted A domain (Figure 7A). In a separate experiment, they replaced part of the interrupted M_S_ domain with the M domain from TioN (another interrupted A domain) (Figure 7B). The observations of these experiments showed that the complete uninterrupted A domain, with the M_S_ domain removed, did not exhibit methylase activity. Moreover, by exchanging the M_S_ with a segment of the methylase domain lacking significant sequence similarity in a naturally interrupted A domain, a new dual-functional A domain was created, retaining its adenylation and methylation activities [76]. Lundy et al. employed a different experimental strategy and observed that when a naturally uninterrupted A domain (Ecm6) was inserted with non-homologous M domains (from KtzH and TioS, respectively), the artificially interrupted A domain also exhibited dual functionality [77] (Figure 7B). Additionally, the linker region between the M domain and the A domain exhibited structured folding without impacting the normal folding of the A domain [56]. Subsequently, researchers achieved A domains with dual interruptions by inserting a main-chain N-methylase domain between a8 and a9 codes and inserting a side-chain S-methylase domain between a2 and a3 codes, enabling the A domain to acquire the functions of adenylation, N-methylation, and S-methylation concurrently [78]. However, an alternative approach involving the simultaneous insertion of two M domains between the interrupted a8 and a9 codes did not show detectable methyltransferase activity, although the adenylation activity and substrate specificity of the A domain remained unaffected [79]. These findings open up new possibilities for exploring the use of auxiliary domains such as MOx, Ox, and KR domains present in NRPS modules to generate interrupted A domains with novel functions. They provide valuable insights into the current research aimed at developing new bifunctional and trifunctional enzymes with adenylation and other activities derived from single-functional A domains in various NRPS systems.

### 3.5. Substitution of C-A Bidomain

Studies have shown that the C domain, which catalyzes the formation of peptide bonds between substrates, has a certain impact on the substrate selection specificity of the A domain. For example, in the A-T double domain of McyB from *Microcystis aeruginosa* PCC 7806, various amino acids such as Leu, Val, Ile, and Tyr can be activated. However, the presence of the C domain on the receptor side biases the substrate preference towards Leu. Similarly, with the C domain present, the McyC module also exhibits a strong preference for the substrate Arg. These observations indicate that the C domain can significantly influence the substrate specificity of the A domain [80].

**Figure 8:**
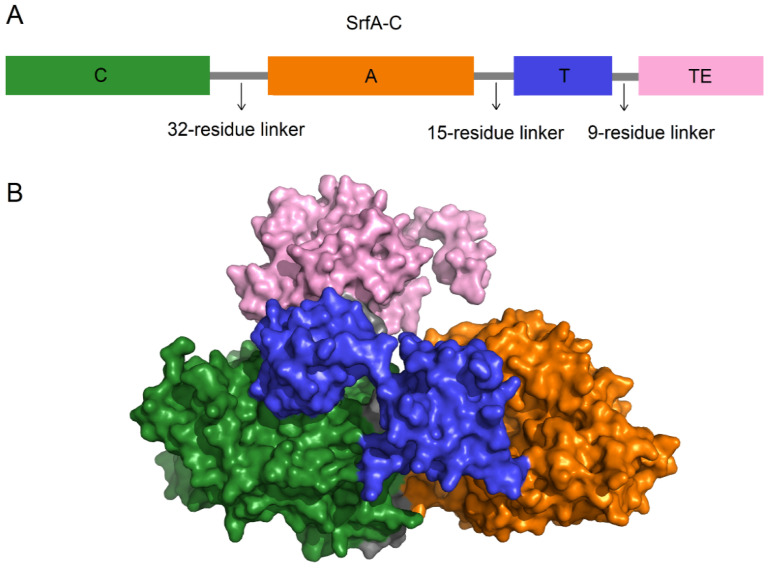
The domain arrangement and overall structure of SrfA-C. (**A**) The domain arrangement of the SrfA-C module: C domain, A domain, T domain, TE domain, and linker regions that connect these domains are represented as green, orange, blue, pink, and gray, respectively. (**B**) The surface representation of the overall structure of SrfA-C (PDB ID: 2VSQ), as well as the colors of the domains and linker regions, are identical to that in (**A**).

Several studies have investigated the domain exchange at the level of the C-A bidomain. Researchers successfully enhanced the production of pyoverdine, a nonribosomal peptide siderophore from *Pseudomonas aeruginosa*, by substituting the C-A bidomain of the second module in PvdD [71]. However, some researchers were unable to create active hybrid enzymes by exchanging heterologous C-A bidomains, suggesting that the substituted C-A bidomains probably are unable to dock properly with the upstream module [81]. Further research on the domain arrangement of the SrfA-C module revealed a linker region containing thirty-two residues between the C and A domains that tightly connects these two domains; as demonstrated in the crystal structure of SrfA-C, the C domain is tightly bound to the A domain to form a stable platform for T domain and TE domain (Figure 8) [82]. Therefore, the influence of the C domain on substrate specificity should also be considered during domain substitution. For example, the substitution of the C4-A10 bidomain in the second module of PvdD involved in pyoverdine biosynthesis from *P. aeruginosa* led to the production of a desired pyoverdine analog, indicating that maintaining the integrity of C-A bidomain may be beneficial [83]. This is also shown by the work of Helge Bode’s group, who introduced a concept called the eXchange unit (XU). The primary forms of XU are A-T-C and A-T-C/E (epimerization domain), allowing exchanges of the C and A domains. The XU can only be applied for exchange when the downstream domains recognize the identical substrate due to the impact of the C domain on substrate specificity [84]. Subsequently, the eXchange unit condensation domain (XUC) was proposed, focusing on overcoming the substrate recognition restriction of the C domain. The form of XUC is C_Asub_-A-T-C_Dsub_, where C_Asub_ represents the acceptor site (approximately the latter half), and C_Dsub_ represents the donor site (approximately the former half) [85]. Recently, another concept, the eXchange unit between T domains (XUT), was established in the form T_1/2_-C-A-T_1/2_ [86]. The discovery of these new exchange sites has expanded the flexibility and diversity of recombining NRPSs.

### 3.6. NRPS Engineering by Whole-Module Rearrangements

**Figure 9:**
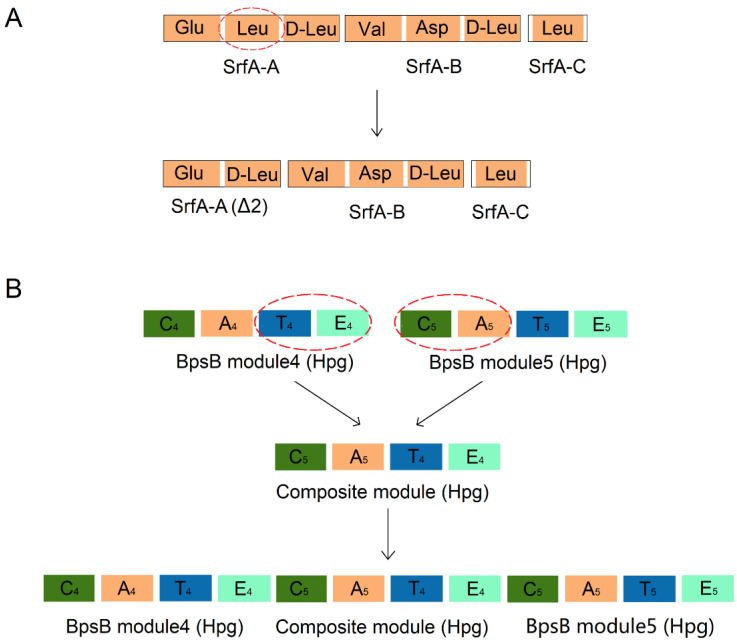
Two typical cases of whole-module rearrangement. (**A**) Deleting the second module in SrfA-A and connecting modules 1 and 3 resulted in the production of a hexapeptide lacking a Leu residue. (**B**) By the insertion of a composite module composed of the T-E bidomain from module 4 and the C-A bidomain from module 5 in BpsB into modules 4 and 5 of BpsB, leading to the production of a novel octapeptide with three Hpg residues. Abbreviation: Hpg: hydroxyphenylglycine.

In addition to the methods of engineering mentioned above, there is another form of engineering at the module level. Engineering of NRPS at the module level to produce novel peptides is mainly achieved through methods such as module swapping, module deletion, and module addition. For instance, within the daptomycin synthetase, module 8 and module 11 are responsible for recognizing D-Ala and D-Ser, respectively. Researchers interchanged these two modules, observing a decrease in the yield of daptomycin analogs compared to the original strain, yet demonstrating the feasibility of module swapping [72]. Additionally, Mootz et al. explored the deletion of an entire module in the NRPS assembly line to generate cyclic peptides with reduced sizes. By removing the SrfA-A2 module that recognizes Leu in the surfactin NRPS and directly connecting module 1 and module 3, the amino acid recognized by the deleted module was removed, and the product was transformed from the original heptapeptide to a hexapeptide variant referred to as ∆2-surfactin (the product of the second module deletion version of SrfA-A) [87] (Figure 9A). 

These methods were also successfully applied in the engineering of NRPS-PKS hybrid modules. Awakawa et al. deleted the coding sequence of the NatD module (domains arranged as C-A-KR-T-TE) from the neoantimycin (tetra-lactone) biosynthetic gene cluster Nat, resulting in a contraction of cycle size and the production of a tri-lactone compound [88]. Additionally, they conducted module extensions to increase the macrocycle size of JBIR-06 (tri-lactone) by inserting the coding gene of the NatD module into the JBIR-06 macrolide biosynthetic gene cluster (Sml cluster). To enable proper expression of the inserted NatD module in SmlC, the C-terminal docking domain of NatC was substituted with the linker and TE domain in the original position of the SmlC module. This substitution maintained module-module interactions, resulting in the production of tetra-lactone products [88]. The NRPS responsible for synthesizing the vancomycin-type glycopeptide antibiotic balhimycin consists of seven modules. Among them, BspA and BspB each contain three modules, while BspC contains only one module. By inserting a hydroxyphenylglycine (Hpg)-selective composite module composed of the T-E bidomain from module 4 and the C-A bidomain from module 5 between modules 4 and 5 of BspB (Figure 9B), researchers detected an octapeptide and a heptapeptide both containing three Hpg residues through product analysis by using HPLC-ESI-MS/MS [89]. Taken together, the successful outcomes of these experiments described above provide valuable examples for further comprehensive investigations into whole-module rearrangements. 

**Table 3:** Several typical engineering methods targeting the A domains.

Engineering Method	Details	Reference
Site-directed mutation	Site-specific mutation of substrate binding site	[26,64,65,66,67,68]
Substitution of A domain	The A domain is replaced by an A domain with alternative substrate-specificity	[69,70,71,72]
Substitution of the recognition subdomain of A domain	Only partial domain sequence associated with substrate recognition is substituted	[74,75]
Domain insertion	Substitution of the domain inserted in interrupted A domain	[76]
	Removal of the inserted domain from the interrupted A domain	[76]
	Inserting domains into non-interrupted A domain	[77]
	Inserting domains at different positions in A domain	[78]
Substitution of C-A bidomain	Substitution of the region, including both C and A domains	[71]
Whole-module rearrangements	Module Replacement	[72]
	Module deletion	[87,88]
	Module extension	[88,89]

## 4. Conclusions

Natural products derived from plants, fungi, and bacteria have long been utilized for treating human diseases [90], playing a crucial role in public health maintenance. A variety of emergency events, such as antibiotic resistance and increased cancer rates, pose a growing threat to human health, making the development of new drugs urgent. Consequently, research on the most diverse and widely distributed natural products, such as NRPs, becomes essential. While most NRPS research has focused on terrestrial organisms, corresponding studies relevant to marine organisms have only been established in recent decades. Marine organisms, owing to their unique ecological habitats and adaptive mechanisms, generate NRPs with unique structures and biological activities. Due to difficulties in marine sample collection, challenges in mimicking the growth environments of marine organisms, and highly variable genomes of these organisms, the exploration of marine organism-derived NRPs as well as the engineering of the corresponding NRPSs are currently insufficient, implying that the field of NRPS research focused on marine organisms holds promising potential for future advancements.

The structural diversity of NRPs is primarily determined by the substrate specificity of the A domain. Therefore, there is a growing emphasis on exploring the modification and engineering of the A domain for producing novel NRPs. This article introduces the substrate recognition and catalytic mechanisms of the A domain, revealing how the A domain adds monomers to the elongating peptide chain. Furthermore, the engineering of A domains using strategies such as mutagenesis of substrate-specificity codes, substitution of domain, domain insertion, and whole-module rearrangements are discussed in detail, presenting advances in these research areas.

After more than two decades of development, both the basic research and engineering of the NRPS system have achieved significant progress. These research observations fully demonstrate the potential applications of NRPSs in the combinatorial biosynthesis of NRPs, further guiding the rational design and engineering of NRPSs and even the de novo design of NRPS assembly lines. Based on the linear catalytic mechanism of NRPS, directed recombination of catalytic modules can theoretically yield peptides with arbitrary sequence combinations. Because A domain is dominant in controlling the sequence of NRP product as illustrated above, the key focus in NRPS assembly line development lies in the engineering of the A domain. 

Due to the complex nature of NRPSs, which possess multiple modules and dynamic function modes, there remains an insufficient comprehension of their internal operational processes and working mechanisms. Enhancing our knowledge of NRPS mechanisms and developing suitable engineering approaches while amalgamating insights from studies on NRPSs from both terrestrial and marine organisms, particularly through the engineering of A domains, holds the potential to empower NRPSs to identify and synthesize novel compounds that have not been naturally discovered. This endeavor is crucial for expanding the diversity within the NRP family and advancing the development of novel pharmaceutical agents. 

## Figures and Tables

**Table 2:** Summary of several types of interrupted A domains with methylase activities.

Product	Interrupted A Domains	Interruption Site	Methylation Type	Reference
Microcystin-LR	McyA (A3-M_b_-A3)	a8, a9	N-methylation	[59]
Pyochelin	PchF (A2-M_b_-A2)	a8, a9	N-methylation	[51]
Kutzneride	KtzH (A4-M_s_-A4)	a8, a9	O-methylation	[55]
Columbamides	ColG (A-M_s_-M_b_-A)	a8, a9	O-methylation, N-methylation	[60]
Micropeptin	McnC (A6-M_b_-A6)	a8, a9	N-methylation	[61]
Thiocoraline	TioS (A3-M_b_-A3) (A4-M_b_-A4)	a8, a9	N-methylation	[53,56]
Thiocoraline	TioN(A-M_s_-A)	a2, a3	S-methylation	[50]
Maremycins	MarQ (A-M_s_-A)	a2, a3	S-methylation	[57]
Thalassospiramide	TtcC (A6-M_b_-A6)	a2, a3	N-methylation	[62]
Thalassospiramide	TtmB (A6-M_b_-A6)	a2, a3	N-methylation	[62]

## Data Availability

No new data were created or analyzed in this study. Data sharing is not applicable to this article.
